# Paralysis of the Lower Extremities

**Published:** 1831

**Authors:** 


### XLIV.

### Paralysis of the Lower Extremities. By Professor Chiappa.

The following case is curious and consolatory. A young female, of slender form but sanguineous temperament, gradually lost the muscular power of the lower extremities—and, in the course of two years, she was totally incapable of moving them. Yet, in this deplorable condition she became, by some *inadvertence*, pregnant, and, in the usual period, gave birth to an infant, without favourable change in the paralysis. She was then transferred to the Clinique of Dr. Chiappa, who noted the following symptoms :—pulse small and hard —frequent cephalalgia—very little sleep —thirst—constipation—violent pain in the left scapula—also in the abdomen, to which she referred the want of sleep. The thighs, but more especially the legs were the seat of acute pain, of a burning kind. All the parts above-mentioned were extremely sensible to the least pressure. The lower extremities were in a state of extreme emaciation.

These symptoms being all taken into consideration, M. Chiappa did not hesitate to attribute them to inflammation of the spinal marrow, extending to the nerves of the abdomen and lower limbs. The treatment was shaped according to this diagnosis, and venesection was practised, while rigid diet, and all the other antiphlogistic measures were put in force. The blood was found to be highly inflamed, as much so as in pleurisy. After four bleedings, leeches were applied to various parts, and in great numbers, on the abdomen, along the spine, to the anus, &c. In the mean time the bowels were kept open by castor oil, while antimonials, acetate of morphine, blisters, digitalis, and even nux vomica were occasionally prescribed. The patient gradually acquired power in the lower extremities, with proportionate mitigation there and in the abdomen. In process of time the patient began to walk, and, at the close of the report, was restored to perfect health.—*Annali Universali*, Feb. 1831.

